# Phosphodiesterase 4 Inhibition Reduces Innate Immunity and Improves Isoniazid Clearance of *Mycobacterium tuberculosis* in the Lungs of Infected Mice

**DOI:** 10.1371/journal.pone.0017091

**Published:** 2011-02-25

**Authors:** Mi-Sun Koo, Claudia Manca, Guibin Yang, Paul O'Brien, Nackmoon Sung, Liana Tsenova, Selvakumar Subbian, Dorothy Fallows, George Muller, Sabine Ehrt, Gilla Kaplan

**Affiliations:** 1 Laboratory of Mycobacterial Immunity and Pathogenesis, The Public Health Research Institute Center at the University of Medicine and Dentistry of New Jersey, Newark, New Jersey, United States of America; 2 National Masan Hospital Clinical Research Center, Masan City Gyeongsangnam-do, South Korea; 3 Celgene Corporation, San Diego, California, United States of America; 4 Department of Microbiology and Immunology, Weill Cornell Medical College, New York, New York, United States of America; Institut Pasteur, France

## Abstract

Tuberculosis (TB) caused by *Mycobacterium tuberculosis* (*Mtb*) is one of the leading infectious disease causes of morbidity and mortality worldwide. Though current antibiotic regimens can cure the disease, treatment requires at least six months of drug therapy. One reason for the long duration of therapy is that the currently available TB drugs were selected for their ability to kill replicating organisms and are less effective against subpopulations of non-replicating persistent bacilli. Evidence from in vitro models of *Mtb* growth and mouse infection studies suggests that host immunity may provide some of the environmental cues that drive *Mtb* towards non-replicating persistence. We hypothesized that selective modulation of the host immune response to modify the environmental pressure on the bacilli may result in better bacterial clearance during TB treatment. For this proof of principal study, we compared bacillary clearance from the lungs of *Mtb*-infected mice treated with the anti-TB drug isoniazid (INH) in the presence and absence of an immunomodulatory phosphodiesterase 4 inhibitor (PDE4i), CC-3052. The effects of CC-3052 on host global gene expression, induction of cytokines, and T cell activation in the lungs of infected mice were evaluated. We show that CC-3052 modulates the innate immune response without causing generalized immune suppression. Immune modulation combined with INH treatment improved bacillary clearance and resulted in smaller granulomas and less lung pathology, compared to treatment with INH alone. This novel strategy of combining anti-TB drugs with an immune modulating molecule, if applied appropriately to patients, may shorten the duration of TB treatment and improve clinical outcome.

## Introduction

Tuberculosis (TB) remains a major global public health problem: Approximately 2 billion people are currently infected, with over 9 million new TB cases and 1.3 million deaths occurring each year [1]. Although the majority of individuals infected by *Mycobacterium tuberculosis* (*Mtb*) do not develop primary active disease, many remain latently infected with a potential for reactivation later in life [2]. Reactivation of latent TB forms a large potential source of new infections. TB chemotherapy is generally efficacious, but current drug regimens require a minimum of six months to achieve cure. Moreover, treatment is often associated with drug toxicities and drug-drug interactions, especially in HIV co-infected patients receiving antiretroviral therapy [3]. If treatment is interrupted or inadequate, the risks of relapse and/or development of drug resistance are high. The availability of effective shorter drug regimens for treatment of TB would greatly reduce these risks and would contribute significantly to improving TB control.

The long duration of therapy required for cure has been attributed to the presence of phenotypically heterogeneous populations of bacilli in infected tissues, including subpopulations of organisms that are not responsive to antibiotics [4]–[7]. These bacillary subpopulations are thought to be in a slow or non-replicating state, rendering them less responsive to the action of anti-TB drugs, many of which were selected for the ability to kill actively growing bacilli [2], [8]. Non-replicating persistence of *Mtb* has been associated with various environmental conditions present in infected host tissues, such as low oxygen tension and limited nutrient availability [9]–[11]. In addition, several studies have implicated reactive nitrogen intermediates (RNI), including nitric oxide (NO), produced by activated macrophages, as an important environmental cue directing the shift of *Mtb* towards dormancy [12]–[14].

Thus, the host immune response may, to some extent, hinder TB treatment by driving a subpopulation of the bacilli into an altered metabolic state, in which they are less responsive to antibiotic killing. Some evidence to support this hypothesis is available from clinical trials using immune modulators as adjunctive therapy in treatment of pulmonary TB [15], [16]. Several reports have described accelerated sputum culture conversion in pulmonary TB patients receiving adjunctive corticosteroids together with antibiotics in comparison with patients who received antibiotic treatment alone [17]–[19]. In one recent study, adjunctive etanercept (soluble TNF-receptor) resulted in reduced time to sputum culture conversion and improved clinical signs in HIV-infected TB patients [20].

To investigate whether immune pressure can affect the responsiveness of *Mtb* to isoniazid (INH) and to test whether immunity can be modified to improve treatment efficacy, we used the mouse *Mtb* aerosol infection model and evaluated the effectiveness of INH treatment in the presence and absence of an immune modulator. For these experiments, we used a novel phosphodiesterase 4 inhibitor (PDE4i) to modulate the host immune response. PDE4 is a member of a diverse family of enzymes, including 11 distinct isoforms that hydrolyze cyclic AMP (cAMP) or cyclic GMP (cGMP) [21]. The distribution of PDE isoforms varies among different tissues and cell types, facilitating the selective inhibition of specific isoforms, as a means of targeting specific cell types and/or activities. cAMP is an important intracellular second messenger which, at increased levels, has anti-inflammatory and tissue protective effects. The specific PDE4i compound used in this study was CC-3052, which increases intracellular levels of cAMP, leading to down-regulation of TNF-α and other inflammatory cytokines in monocyte/macrophages [22]–[24]. CC-3052 is water soluble and has been shown to be non-toxic, non-mutagenic and non-teratogenic [23]. The drug demonstrated little or no measurable effect on T cell activation [25], [26], which is consistent with the finding that activation of T cells is regulated by the isozyme PDE7 but not PDE4 [27]. Thus, CC-3052 would not be expected to cause generalized immune suppression. We hypothesized that immune modulation with CC-3052 would alter the intracellular environment within the infected macrophage, so that a greater proportion of the bacilli would remain in a more metabolically active state and would retain their responsiveness to INH. The effects of CC-3052 on host global gene expression, induction of cytokines, and T cell activation in the lungs of infected mice were evaluated. Our results suggest that selective modulation of the innate immune response with a PDE4i can favorably alter the kinetics of INH-mediated bacillary killing and may enhance the efficacy of TB drug therapy.

## Results

### Effect of CC-3052 on INH-mediated killing of *Mtb* in lungs of infected mice

**Figure 1:**
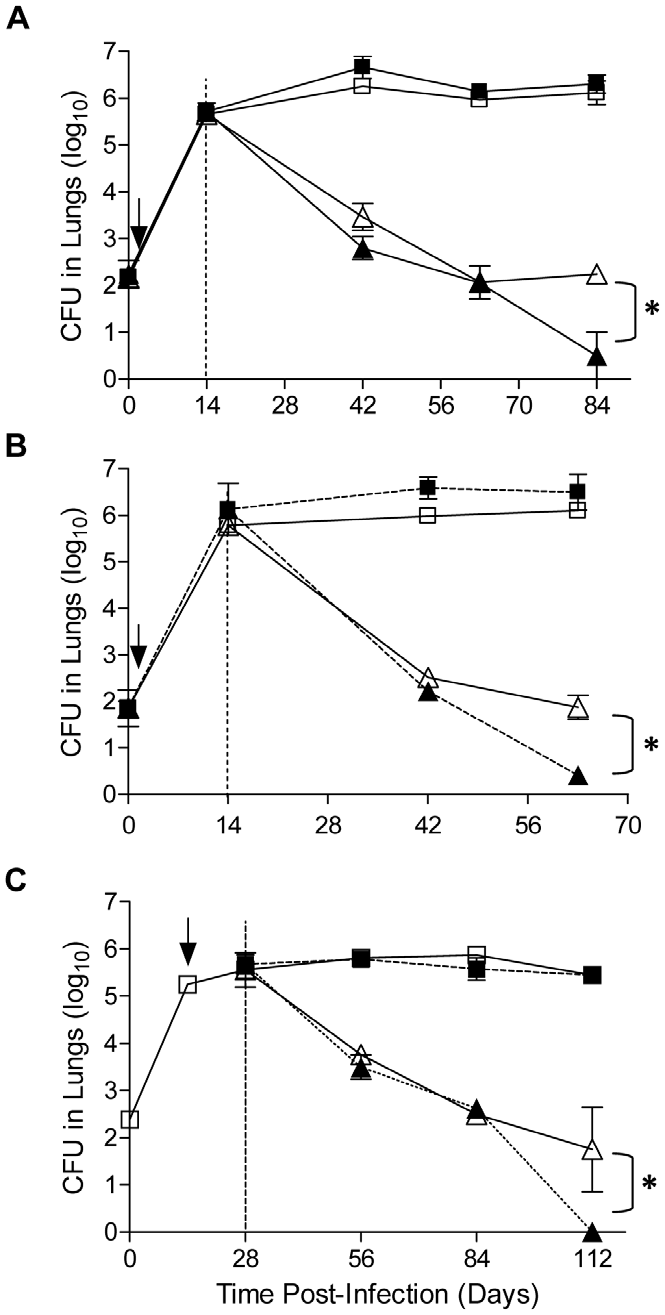
Effect of CC-3052 on *M. tuberculosis* bacillary load in lungs during INH treatment. B6D2F1 mice were infected by low dose aerosol (100–200 cfu) with *M. tuberculosis* clinical isolates CDC1551 (A and C) or HN878 (B) [28]. CC-3052 treatment was initiated on day 1 (A and B) or day 14 (C) (arrow) after infection, and INH treatment was initiated at 14 days (A and B) or day 28 (C) post-infection (dotted line). Results are the mean CFU±SD of 2–6 independent experiments for CDC1551 (A); 2 experiments for HN878 (B) and 2 experiments for CDC1551 (C) (3–4 mice/group/experiment). Treatment groups: Untreated (open square); CC-3052 treated (filled square); INH treated (open triangle); CC-3052+INH treated (filled triangle). * Represents a statistical significant difference (*P*<0.05) between INH and CC-3052+INH treated mice.

For these studies, we selected two clinical strains of *Mtb* that differ in their abilities to induce an immune response in mice and in human monocytes [28]. Although the growth curves do not differ substantially in mice, CDC1551 promotes a strong, early Th1 response and is less virulent in mice, while HN878 is less immunogenic and highly virulent, causing earlier death of infected mice. Mice were infected by low dose aerosol infection with *Mtb* strain CDC1551 or HN878 and treated with CC-3052 from day 1 post-infection; bacillary growth, measured as colony forming units (CFU) in the lungs, was evaluated over time (Figure 1). The numbers of bacilli in the lungs of CDC1551-infected mice treated with CC-3052 were similar to those observed in untreated control infected mice, both of which stabilized by about 28 days post-infection (Figure 1A). Throughout the experiment (84 days), there were no significant differences in bacterial loads between CC-3052-treated and untreated mice (*P* = 0.882), suggesting that CC-3052 treatment of the mice did not accelerate the growth of *Mtb* in the lungs. INH treatment, initiated on day 14 post-infection, initially reduced the CFU efficiently in both experimental groups, but by 63 days post-infection, the bacterial loads in the lungs of mice treated with INH alone stabilized at about 2 log_10_. These results resemble the characteristic bi-phasic killing curve that was seen in early studies of INH killing in the mouse [29], [30]. In contrast, the CFU in the lungs of mice co-treated with INH plus CC-3052 continued to be cleared and at 84 days were significantly lower in numbers than those in mice treated with INH alone (*P* = 0.016). Plating of lung homogenates from all experimental groups on INH-containing solid medium yielded no colonies, indicating that the residual bacilli in the lungs resulted from antibiotic tolerance rather than acquired resistance to INH. CC-3052 treatment alone had no impact on the kinetics of *Mtb* growth in broth culture (not shown), demonstrating that the drug has no direct bactericidal/static effect. *Mtb*-infected mice treated with CC-3052 showed no significant reduction in body weight during the course of the experiment, as compared to untreated control mice (not shown). A similar pattern of bacillary clearance was observed in mice infected with *Mtb* strain HN878, indicating that the effects of CC-3052 are not *Mtb* strain-specific (Figure 1B). Shifting the timing of treatment also did not alter the ability of CC-3052 to impact bacillary clearance in the lungs. When CDC1551-infected mice were treated with CC-3052 and INH beginning on day 14 and day 28 post-infection, respectively, *Mtb* killing by INH alone did not slow until 84 days post-infection; the CFU numbers continued to decline in co-treated mice up until day 112 (Figure 1C).

We evaluated the impact of varying the dose of CC-3052 treatment (5 mg/kg, 25 mg/kg, 100 mg/kg) on the kinetics of *Mtb* growth in the lungs of infected mice and found no differences in the CFU over 28 days (not shown). Importantly, administration of high dose CC-3052 (100 mg/kg), which is above the pharmacologically active dose (Celgene Corporation, unpublished data), did not adversely affect the ability of the animals to control *Mtb* growth in the lungs.

### Effect of CC-3052 on histopathology of *Mtb*-infected mouse lungs

**Figure 2:**
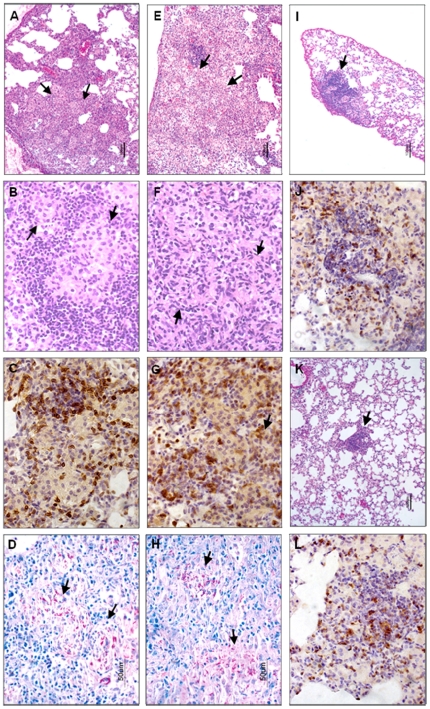
Effect of CC-3052 on histopathology of CDC1551-infected mouse lungs with or without INH. Treatment with CC-3052 was initiated from day 1 and INH was administered from day 14 after infection. Sections of lungs of mice infected for 42 days are shown. The arrows show accumulation of macrophages (H&E: A, B, E, F, I, K) or the presence of AFB (ZN: D, H). CD3^+^ T cells are brown (anti CD3 monoclonal Ab: C, G, J, L). Untreated control (A–D); CC-3052-treated (E–H); INH-treated (I, J); CC-3052+INH treated (K, L). Magnification ×4: A, E, I and K; ×40: B–D, F–H, J and L.

Specimens of lung tissue were collected from CDC1551-infected mice treated with INH from day 14 and/or CC-3052 from day 1; these were prepared for histopathologic and immunohistologic analysis. Examination of H&E stained sections from lungs collected during the first 4 weeks of infection showed minimal differences among the different experimental groups (not shown). At 42 days post-infection, the lungs from CC-3052-treated mice showed somewhat less organized granulomas than those in control untreated mice, but the differences were not striking (Figure 2). The lesions in the lungs of untreated mice contained small aggregates of macrophages (arrows) surrounded by large numbers of lymphoid cells (Figure 2A and B). In the lesions of these mice, CD3^+^ staining cells were distributed predominantly around the aggregates of macrophage and accounted for a minority of lymphoid cells seen in the granulomas (Figure 2C). An average of 171±26 CD3^+^ T cells were seen in each ×40 field of granuloma. The macrophages contained single or small clusters of acid-fast bacilli (AFB) (Figure 2D arrows). By comparison, in the granulomas of CC-3052-treated mice, more diffuse macrophage aggregates and more evenly dispersed lymphoid cells were seen (Figure 2E and F). The number of CD3^+^ T cells was similar to that seen in control untreated mouse lungs, with an average of 156±22 CD3^+^ T cells per ×40 field of granuloma (Figure 2G). In CC-3052-treated animals too, the macrophages contained single or small clusters of AFB (Figure 2H). In contrast, the lungs of mice treated with INH alone contained condensed small granulomas with many lymphoid cells (Figure 2I), with few CD3^+^ T cells (Figure 2J) and few AFB (not shown). CC-3052 plus INH treatment at 42 days resulted in even fewer and smaller granulomas (Figure 2K and L), compared to those of mice treated with INH alone, and a further reduction in the numbers of visible AFB (not shown).

Morphometric analysis of the extent of the granulomatous infiltration in the lungs of infected mice showed differences in the number of granulomas per cm^2^ and in the percent of area of the lung involved in granuloma formation among the different treatment groups (mean of 3 experiments). At 35 days post-infection, the average numbers of granulomas per cm^2^ in the lungs of the different treatment groups were: controls, 91.2±21.0; CC-3052, 89.1±28.0; INH, 36.7±8.4; and INH plus CC-3052, 20.6±11.3 (Figure S1). The average area of the lung parenchyma occupied by granulomas was: controls, 25.9±9.1%; CC-3052, 21.2±8.3%; INH, 1.7±0.3%; and CC-3052 plus INH, 1.7±1.0%. By 63 days post-infection, the extent of pathology was reduced in all experimental groups. The number of granulomas and the area occupied in the lungs had declined to: controls, 41.8±19.6 granulomas per cm^2^ and 14.3±6.2% of the area of the lung involved; for CC-3052, 67.1±0.1 granulomas per cm^2^ and 8.6±0.2% of the area of the lung involved. In the INH-treated animals, the granulomatous response involved: INH, 11.1±2.9 granulomas per cm^2^ and 1.0±0.3% of the area, respectively; versus INH plus CC-3052, 3.4±0.4 granulomas per cm^2^ and 0.2±0.1% of the area, respectively. Thus, the combination of CC-3052 plus INH treatment resulted in significantly lower (*P* = 0.02) lung involvement (percent of the lung with pathology) compared to INH alone (Figure S1).

### Effect of PDE4 inhibition by CC-3052 treatment on host gene expression in the lungs of *Mtb*-infected mice

**Figure 3:**
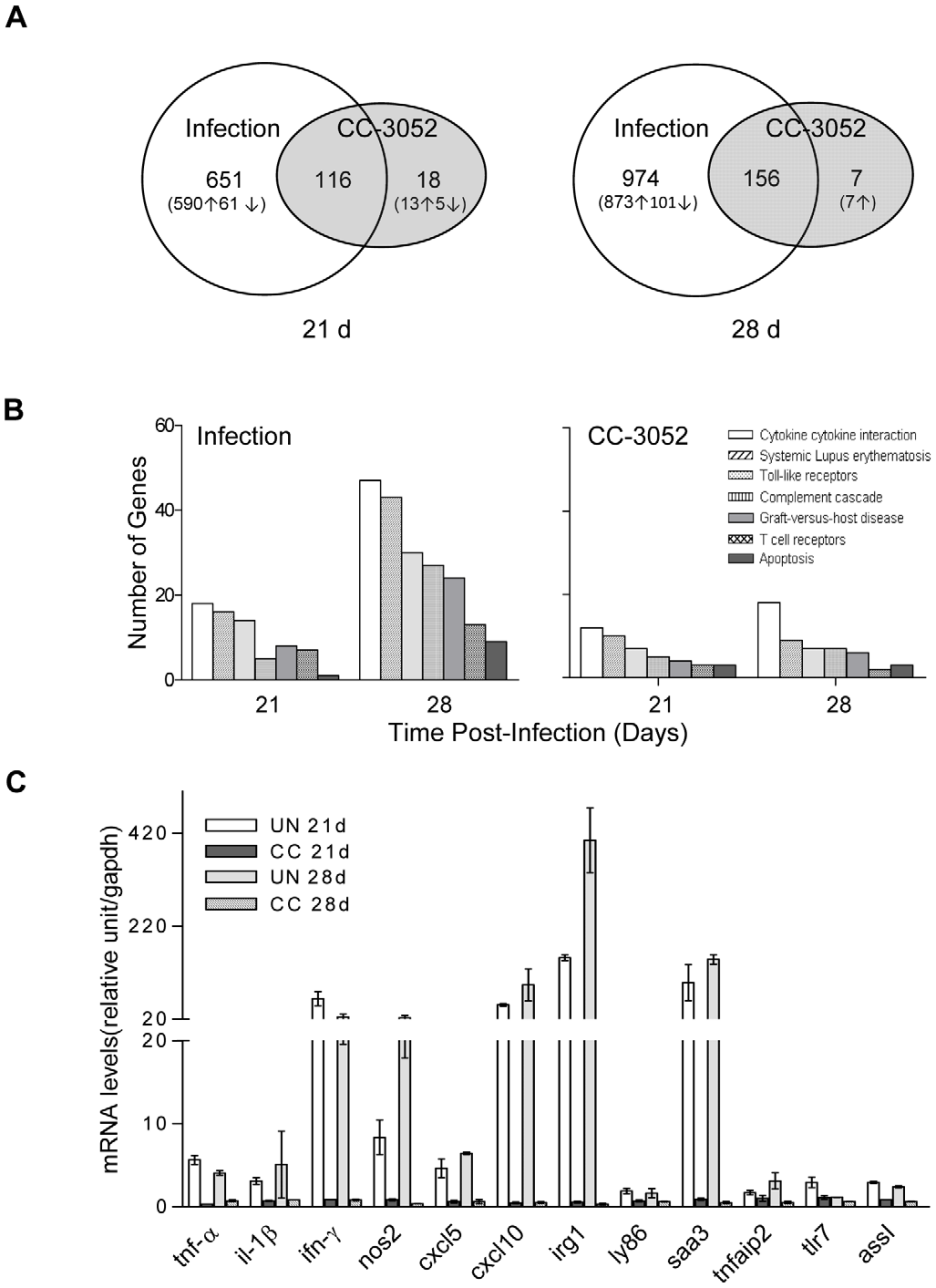
Analysis of transcriptional profile by gene ontology and cytokine mRNA levels by qPCR. Lungs from CDC1551-infected mice treated with CC-3052 or untreated at 21 or 28 days post-infection were used to prepare mRNA. (A) Venn diagram of genes differentially expressed in lungs of uninfected versus *Mtb*-infected mice (open) or *Mtb*-infected mice treated with CC-3052 versus untreated (shaded) at 21 and 28 days post-infection. The number of genes was calculated based on ≥1.5 fold change and P<0.05 cutoff using Partek Genomic Suite. Four independent arrays per group were performed. (B) Gene ontology of global response and immune response to infection and to CC-3052 treatment in mouse lungs was analyzed using Pathway Express. (C) mRNA levels of selected cytokine genes using qPCR to validate DNA array data listed in Table S1. PCR was performed with the same RNA samples in duplicate independently from 4 mice per experimental group. Results of the qPCR are represented as mean ± SD. The gene ID numbers and gene definitions may be viewed at http://www.ncbi.nlm.nih.gov.

The impact of PDE4 inhibition on gene expression in the lungs of *Mtb*-infected mice was evaluated by Affymetrix DNA array. Mice were infected with CDC1551, with and without CC-3052 treatment initiated on day 1. Total RNA was isolated from the lungs at 14, 21, 28, and 42 days post-infection to follow temporal changes in global gene expression. At 14 days post-infection, relatively few genes (45) were differentially expressed in response to infection in the absence of PDE4i, based on Significance Analysis of Microarrays (SAM) with a cut-off of ≥1.5-fold differences using Partek Genomics Suite (Table 1). The number of differentially expressed genes increased by 21 days (767) and was maximal at 28 days (1,130), declining again by 42 days (693) post-infection (Table 1). This temporal pattern is consistent with what is known about the host immune response in murine pulmonary TB infection, which involves initial recruitment of leukocytes to the site of infection and activation of the pro-inflammatory response, followed by dampening of the early response, as granulomas are established and the infection matures into a chronic steady-state [31], [32]. The functional categories of host genes most affected by *Mtb* infection included innate immune response, T cell activation, antimicrobial response, apoptosis and metabolism, based on analysis by Pathway Express (Figure 3B), consistent with a previously published study of the murine transcriptional response to *Mtb* [33].

**Table 1:** Number of Differentially Regulated Genes.

	14 d	21 d	28 d	42 d
A. Gene sets regulated by Mtb infection				
Up	41	700	1025	683
Down	4	67	105	10
B. Gene sets regulated by CC-3052 treatment				
Up	4	20	5	15
Down	33	114	158	28

Gene expression was analyzed from total RNA of uninfected and *Mtb*-infected mouse lungs, treated or not treated with CC-3052. Results are representative of 4 independent arrays per group and presented as relative gene expression. Number of differentially regulated genes was determined based on cutoff of P<0.05 and ≥1.5 fold change using Partek Genomics Microarray.

Treatment of infected mice with CC-3052 affected the expression of a accumulative total of 186 genes over the course of infection, of which 164 were down-regulated and 22 were up-regulated (≥1.5 fold), as compared to no treatment (data not shown). The temporal changes in gene expression induced by CC-3052 were similar to those due to infection, with relatively minor changes at 14 days (4 genes up-regulated; 33 genes down-regulated), increasing effects at day 21 (20 up; 114 down) and day 28 (5 up; 158 down), and fewer genes differentially expressed at 42 days (15 up; 28 down) post-infection (Table 1). The numbers of genes differentially expressed by *Mtb* infection alone or CC-3052 treatment at 21 and 28 days post-infection were compared using Ingenuity Pathway Analysis (IPA), which includes only annotated genes. Of the genes differentially expressed in response to CC-3052 treatment, 86.6% (116/134) and 95.7% (156/163) overlapped with those whose expression was affected by *Mtb* infection (Figure 3A). Many of the genes differentially expressed by CC-3052 treatment were involved in the host innate immune response, for example: innate immunity (*cd*68, *clec*4e, *clec*5a, *clec*7a, *clec*12, *ly*86, *irg*1, *tlr*7, *c1qa*, *c1qb*, *c1qc*), inflammatory response (*ccl*3, *ccl*8, *cxcl*5, *cxcl*9, *cxcl*10, *ccr*5, *cxcr*6, *ifn*-γ, *il*-1α, *il*-1β, *tnf*-α, *tnfaip*2, *tnip*3), immune regulation (*aoah*) and apoptosis (*bub*1) (Table S1). In general, treatment with the PDE4i resulted in down-modulation of a subset of the same genes that were up-regulated by infection. The genes that were differentially expressed in response to CC-3052 treatment belonged to pathways regulating cytokine-cytokine receptor interaction, Toll-like receptor signaling, complement and coagulation cascades, graft-versus-host disease, T cell receptor signaling, and apoptosis (Figure 3B).

Quantitative PCR (qPCR) was used to confirm the differential expression of a subset of genes associated with the pro-inflammatory response to *Mtb* infection (Figure 3C). TNF-α, IL-1β, NOS2 and IFN-γ play a central role in the activation of macrophages and are essential for host control of *Mtb* infection [32], [34]. TNF-α, IL-1β and NOS2 are produced by macrophages in response to infection with *Mtb*, while IFN-γ is produced by dendritic cells and various lymphoid cells, including lymphoid cells of the innate immune response. Using the same mRNA pools from the CC-3052 treated and untreated mice at 21 and 28 days post-infection, we evaluated the impact of PDE4i treatment on the expression of these genes. We found that *Mtb* infection significantly increased the expression of *tnf-*α, 2.49/3.01; *ifn-*γ, 2.45/4.11; *il*-1β, 2.76/3.43; and *nos*2, 2.69/5.98 at 21 and 28 days post-infection respectively (differential expression ≥1.5 fold, *P*<0.05). In contrast, CC-3052 treatment resulted in decreased gene expression: *tnf-*α, −1.63/−1.50; *ifn-*γ, −1.64/−1.54; *il-*1β, −1.67/−1.58; and *nos*2, −1.68/−1.84 at 21 and 28 days post-infection respectively (Figure 3C). In addition, we used qPCR to compare the relative expression of a number of other genes which are known to be regulated by TNF-α, including those encoding chemokines (*cxcl*5, *cxcl*10), TNF-α induced protein 2 (*tnfaip*2), and argininosuccinate synthetase 1 (*ass*1) [35], [36]. All of these genes were up-regulated in response to *Mtb* infection and down-regulated by CC-3052 treatment at both 21 and 28 days post-infection, confirming that TNF-α is a target of PDE4 inhibition. We also examined the expression levels of a number of genes which are known to play important roles in innate immunity, including Toll-like receptor 7 (*tlr*7), serum amyloid A3 (*saa*3), immune responsive gene (*irg*1), and lymphocyte antigen 86 (*ly*86) [33], [37]–[41]. These genes were similarly up-regulated by *Mtb* infection and down-regulated by CC-3052 treatment (Figure 3C).

One potential explanation for the impact of CC-3052 treatment on global gene expression in the infected mice may be that PDE4 inhibition is causing changes in the relative distributions of cell types in the lungs. To test this possibility, single cell suspensions were prepared from the lungs at 28 days post-infection and used to analyze the cellular composition of the tissues by flow cytometry. Results of this analysis showed that treatment with CC-3052 did not affect the relative abundance of CD4^+^ and CD8^+^ T cells and monocyte/macrophages (CD14^+^) in the lungs (Figure S2A and B). In addition, by staining the cell suspensions for intracellular IFN-γ and analyzing by flow cytometry, we showed that the percentages of CD4^+^ and CD8^+^ T cells producing IFN-γ in the lungs were unaffected by CC-3052 treatment (Figure S2C).

### Effect of CC-3052 treatment of mice on *Mtb-*induced spleen T cell activation

**Figure 4:**
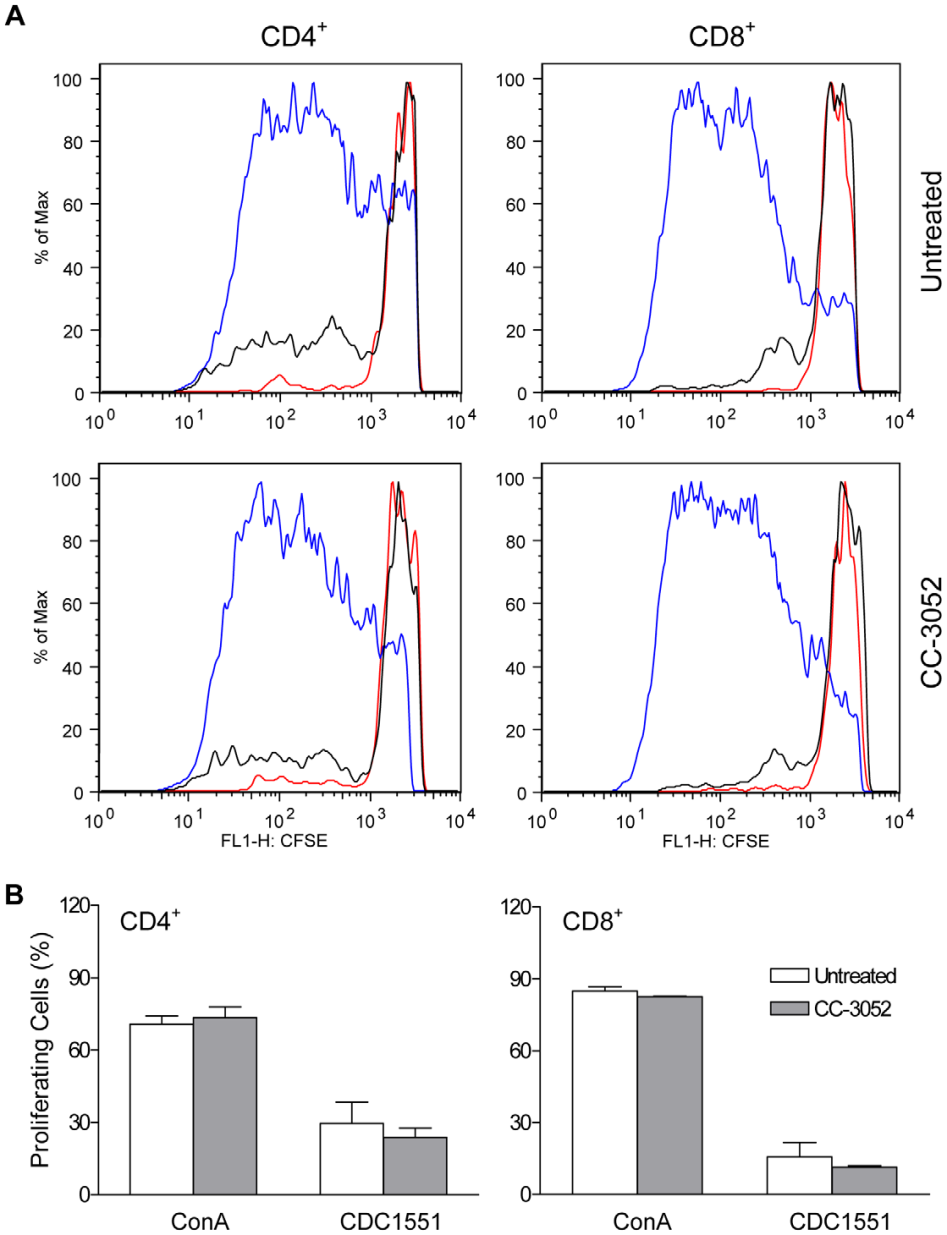
Effect of CC-3052 on proliferation of spleen T cells. Spleen cells from CDC1551-infected mice treated with CC-3052 or untreated were harvested at 42 days post-infection, labeled with CFSE and stimulated in culture for 6 days with heat-killed CDC1551 (black), ConA (5 µg/ml) (blue), or left unstimulated (red). Cells were stained with anti-CD4 and anti-CD8 antibodies. Proliferation was measured as a reduction in CFSE fluorescence intensity. (A) Proliferating lymphocytes are displayed as histograms. One representative mouse per group is shown. (B) The percent of CD4^+^ and CD8^+^ proliferating cells from 3 mice per group are presented as a mean ± SD.

The impact of CC-3052 on *Mtb*-specific activation of CD4^+^ and CD8^+^ T cells in chronically infected mice (42 days), at the time when acquired immunity is fully established, was evaluated. Single cell suspensions prepared from spleen were stimulated *ex vivo* with ConA or *Mtb* CDC1551 for 6 days, and T cell proliferation was measured by the carboxyfluorescein succinimidyl ester (CFSE) flow cytometry assay. The capacity of CD4^+^ and CD8^+^ T cell to proliferate in response to ConA or CDC1551 stimulation was similar in spleen cells from the infected mice with or without CC-3052 treatment (Figure 4). Our observations suggest that CC-3052 does not interfere with T cell activation, consistent with published reports [25], [26].

### Effect of CC-3052 treatment on monocyte/macrophage activation

**Figure 5:**
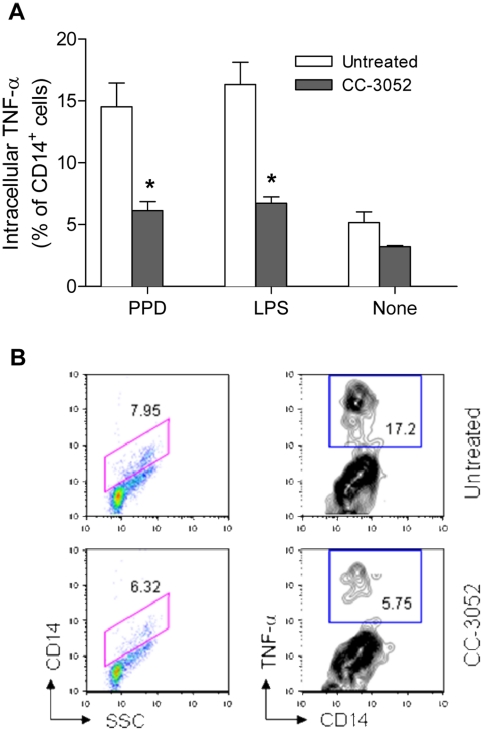
Effect of CC-3052 on intracellular TNF-α in spleen cells. (A) Percent of CD14^+^ spleen cells producing intracellular TNF-α at 21 days post-infection after *in vitro* stimulation with PPD (*P* = 0.05), LPS (*P* = 0.04), or None (unstimulated). Data shown are the mean ± SD of three mice per time point. Untreated mice (open columns) and CC-3052-treated mice (closed columns). * Represents a statistically significant difference (*P*<0.05) between untreated controls (UN) and CC-3052 (CC) treated mice. *P* values were calculated using the unpaired *t* test. (B) Representative contour density plot for PPD stimulated cells.

To evaluate the impact of CC-3052 on monocyte/macrophage activation, the ability of CD14+ cells to produce TNF-α in response to *ex vivo* stimulation was assessed by flow cytometry. Single cell suspensions from spleens of untreated and CC-3052-treated mice were collected at 21 days post-infection and stimulated *ex vivo* for 6 h by PPD or LPS. The percentage of CD14^+^ cells producing TNF-α was significantly lower in spleens from CC-3052 treated mice than those from untreated infected control mice following *ex vivo* stimulation with PPD (*P = *0.05) or LPS (*P = *0.04) (Figure 5A and B). These data confirm that CC-3052 targets monocyte/macrophage function and support the results of the gene expression profiling, indicating that treatment of *Mtb*-infected mice with the PDE4i causes modulation of innate immunity. Taken together, the results of the *ex vivo* stimulation experiments suggest that CC-3052 modulates the innate immune response, without affecting T cell activation, indicating that the drug is not generally immune suppressive, as previously described [25], [26].

### Effect of anti-TNF-α antibody (TN3-19.12) on the mouse response to *Mtb* infection

**Figure 6:**
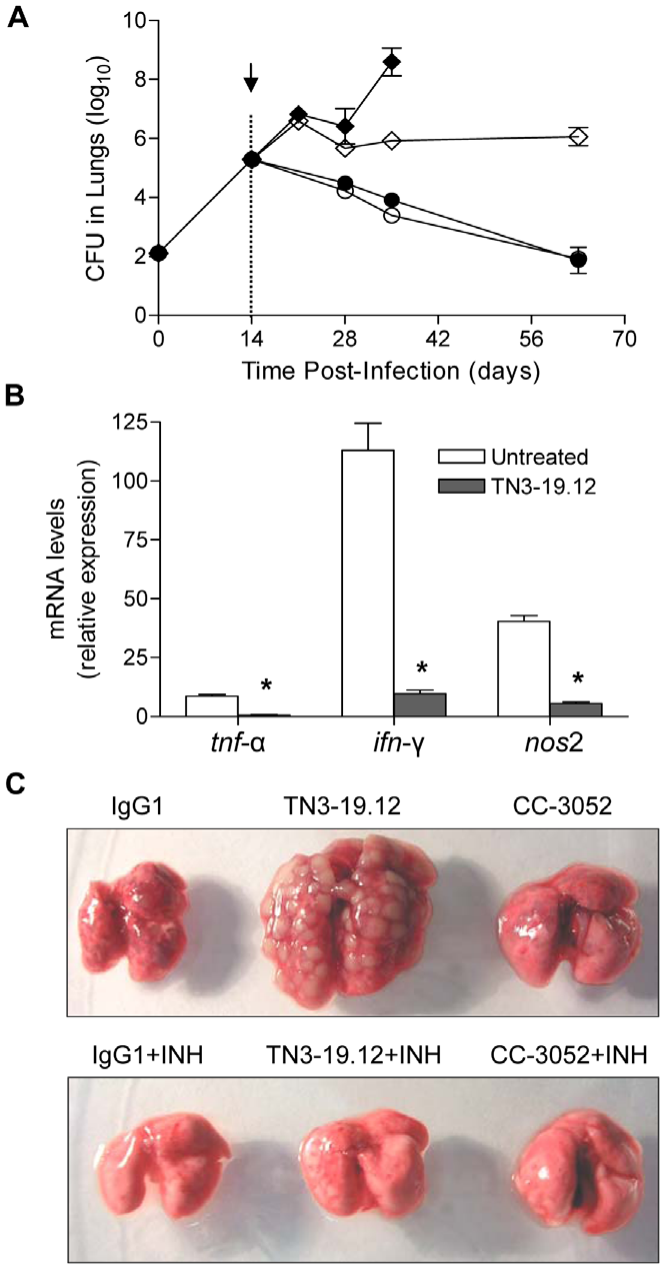
Effect of TN3-19.12 on bacillary load, cytokine production and gross pathology. IgG1 (arrow), TN3-19.12 (arrow) and/or INH treatment (dotted line) were initiated at 14 days post-infection. (A) Bacillary load in mouse lungs. Open diamond, IgG1; Closed diamond, TN3-19.12; Open circle, INH; Closed circle, TN3-19.12+INH, Results are the mean ± SD of 3–4 mice per time point per treatment group. (B) qPCR on mouse lungs collected 28 days post-infection. Results are the mean ± SD of 3–4 mice per time point per treatment group; IgG1- open bars, TN3-19.12- filled bars. * Represents a value of *P*<0.05 for TN3-19.12 relative to the IgG1 treated mice (control). (C) Gross appearance of the lungs from mice treated with IgG1 (control), TN3-19.12 or CC-3052, with or without INH co-treatment, at 35 days post-infection.

As a comparison with the selective inhibition of monocyte/macrophage TNF-α induced by treatment of mice with CC-3052, we evaluated the impact of treatment with a monoclonal anti-TNF-α neutralizing antibody (TN3-19.12) on *Mtb* infection of mice [42]. Mice infected with CDC1551 were treated with IgG1, TN3-19.12 and/or INH starting at 14 days post-infection. In response to treatment with TN3-19.12 alone, *Mtb* continued to grow logarithmically in the lungs, reaching levels of 9 log_10_ CFU at 35 days post-infection, after which none of the mice in this treatment group survived (Figure 6A). In contrast, the infection was controlled in lungs of untreated mice, achieving a steady state of about 6 log_10_ bacilli from 28 days post-infection to the end of the experiment (Figure 6A). In mice treated with INH, bacillary loads decreased similarly in both the TN3-19.12 and control IgG1 co-treated animals. In addition, treatment of mice with TN3-19.12 alone resulted in progressive weight loss. By 14 days of treatment (28 days post-infection), mice had lost more than 20% of their body weight and were moribund (not shown). The combination of high bacillary burden and severe weight loss in the TN3-19.12-treated animals was associated with rapid mortality (within 3 weeks post-initiation of treatment). In contrast, mice in the other treatment groups gained weight similarly over time. The expression levels of genes encoding TNF-α, IFN-γ and NOS2 in the lungs of mice treated with TN3-19.12 at 28 days post-infection, were significantly reduced compared to the control IgG1 treated animals (Figure 6B).

### Impact of TN3-19.12 on gross appearance and histopathology of CDC1551-infected mouse lungs

The effect of TNF-α inhibition with TN3-19.12 on gross lung appearance was compared to the effect of inhibition with CC-3052. Lungs from CDC1551-infected mice treated from day 14 with IgG1 (control), CC-3052, or TN3-19.12 with or without INH co-treatment were isolated at 35 days post-infection. The lungs harvested from CC-3052 treated mice were slightly enlarged compared to those from IgG1 control mice (Figure 6C). In contrast, the lungs from TN3-19.12-treated mice were strikingly enlarged, with numerous large granulomatous lesions not seen in the IgG1 control or CC-3052-treated mouse lungs (Figure 6C). INH treatment resulted in a clear reduction in lung size and lesion size in all treatment groups.

**Figure 7:**
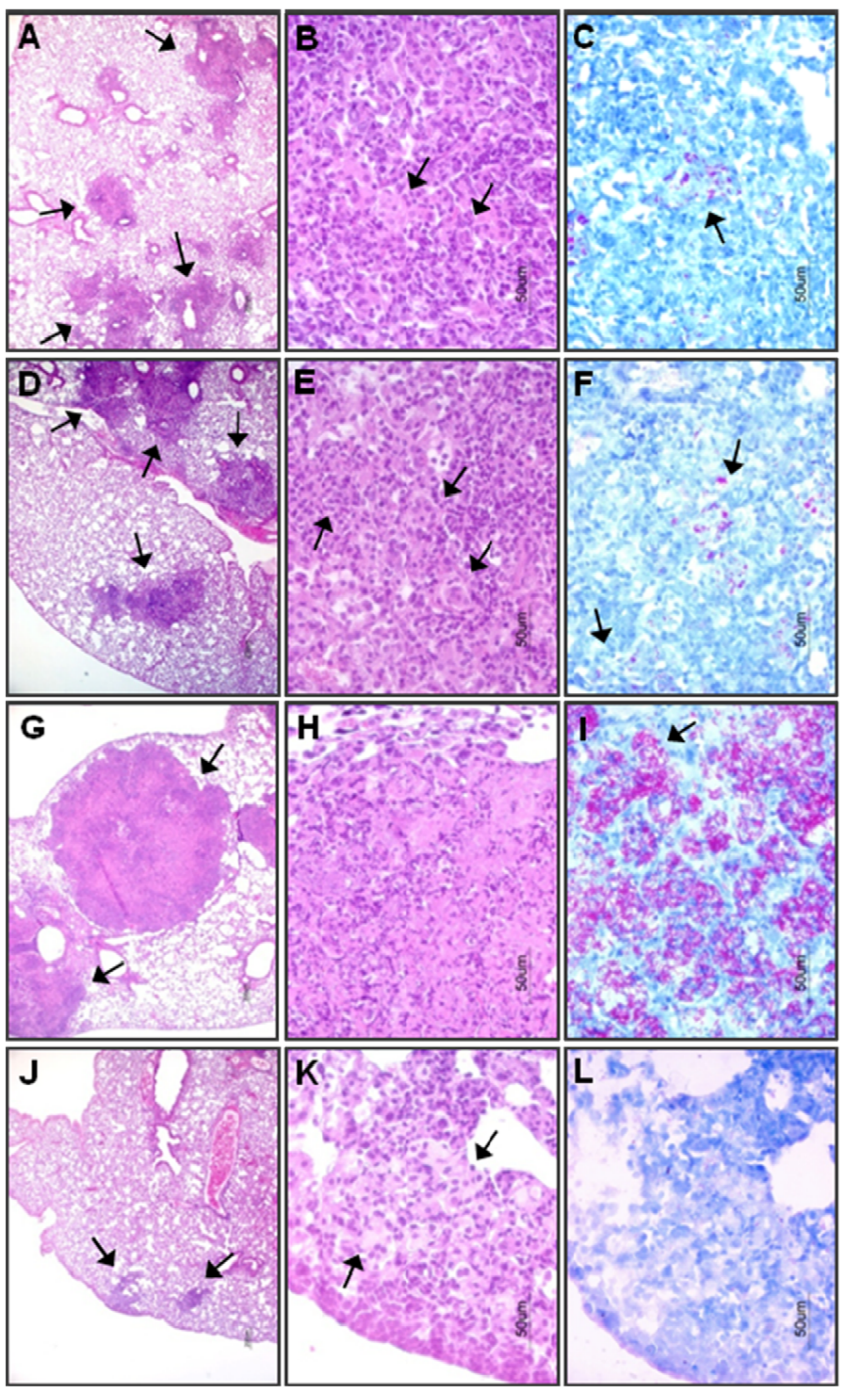
Effect of CC-3052, TN3-19.12, or INH on histopathology of lungs from CDC1551-infected mice. Treatment with IgG1, monoclonal anti-TNF-α antibody (TN3-19.12), CC-3052 and/or INH was initiated at 14 days post-infection and lungs were harvested at 35 days post-infection and histologic analysis was done. (A–C) IgG1-treated (control); (D–F) CC-3052-treated; (G–I) TN3-19.12 treated; (J–L) TN3-19.12+INH treated. Left panel, H&E ×4; Middle panel, H&E ×40; Right panel; ZN ×40. Arrows indicate granulomas (left panel), macrophages (middle panel) and AFB (right panel).

Histopathologic evaluation of lungs at 35 days post-infection in both control and CC-3052-treated mice revealed similar, well organized granulomas, with small clusters of AFB clearly visible (Figure 7A, C, D and F). The granulomas of CC-3052 treated mice were somewhat less compact, with many macrophages and more evenly distributed lymphocytes than those in control animals (Figure 7B and E). In contrast, mice treated with TN3-19.12 had large granulomas with many polymorphonuclear neutrophils (PMNs) (Figure 7G) and extensive necrosis (Figure 7H). Extremely high numbers of AFB were detected in the granulomas of these mice (Figure 7I). By comparison, co-treatment with TN3-19.12 plus INH resulted in smaller, well-organized lung granulomas, with higher numbers of lymphocytes and few to no AFB (Figure 7J, K, and L).

## Discussion

We have shown that modulation of the immune response in CDC1551 or HN878-infected mice with the PDE4i CC-3052 improves INH-mediated *Mtb* killing in the lungs. This was demonstrated by the clearance of *Mtb* in INH plus CC-3052 co-treated animals, which continued to decline up to the end of the infection, in contrast with clearance during the first 2 months of treatment in INH only treated mice, where *Mtb* killing reached a plateau before the end of the experiment. Thus, while the rate of bacillary clearance was similar in both treatment groups, the extended duration of killing in the CC-3052 co-treated mice resulted in a difference of approximately 2 log_10_ in CFU, as compared to INH treatment alone, at later time points. The continued clearance of bacilli is similar to the impact of adding pyrazinamide (PZA), which is more active against non-replicating bacilli, to an INH monotherapy regimen, as described in previous studies on treatment of *Mtb*-infected mice [30], [43]. Although it is not clear whether CC-3052 co-treatment can improve the efficacy of multidrug TB therapy, the use of a host-targeted drug has a considerable benefit because *Mtb* cannot develop genotypic resistance to a drug that targets host function. In our study, we did not evaluate the ability of co-treatment with CC-3052 plus INH to achieve sterilization of the bacilli. Larger and more extensive experiments to test whether these mice can reactivate the infection upon discontinuation of treatment and general immune suppression studies are needed to answer this question.

Treatment with CC-3052 alone had no impact on *Mtb* growth in vitro or in vivo, indicating that the drug itself is not bactericidal/static and does not enhance bacillary replication. Rather, we suggest that down-modulation of the host innate immune response may have dampened some environmental signals that normally drive a subset of bacilli into a less metabolically active state, in which they are not responsive to INH-mediated killing. This would explain why the enhanced efficacy of INH-mediated killing by CC-3052 was manifested only at later time points, when the rate of bacillary clearance mediated by INH alone slowed down, resulting in the bi-phasic killing curve. This hypothesis must now be confirmed in experiments to evaluate the physiologic state of the bacilli in the lungs of mice treated with CC-3052.

The results of the host gene expression studies showed that treatment with CC-3052 resulted in down-regulation of a subset of immune response genes that were up-regulated in response to *Mtb* infection. These included many genes that are involved in innate immunity and inflammation. This result is consistent with our observations that T cell activation was unaffected in mice treated with CC-3052 alone, and that these mice were fully capable of controlling the infection by 3–4 weeks, the time at which the adaptive immune response is optimally induced. It was interesting to find that the expression of *nos*2, which is responsible for macrophage production of NO, was significantly lower in the lungs of mice treated with CC-3052 at 21, 28 and 42 days post-infection. As NO has been implicated in the induction of *Mtb* non-replicating persistence [12], [13], [31], it is intriguing to speculate that reduction of NO by CC-3052 treatment may partially explain the improved bacillary clearance. This possibility is supported by the recent identification of a novel antimicrobial primarily active against non-replicating organisms, which targets *Mtb* dihydrolipamide acyltransferase (DlaT), an enzyme necessary for resistance to RNI-induced stress [44]. It is also of interest to note that *Mtb* secretes endogenous cAMP, shown to increase the levels of host TNF-α production [45]. Agarwal et al suggest that their findings may provide a mechanism by which *Mtb* itself may contribute towards eliciting granuloma formation as part of its survival program.

Small molecule PDE4i penetrate the plasma membrane and act to increase cAMP levels within the cells [22], reducing production of TNF-α and other innate immune mediators within the macrophage. Thus, treatment with CC-3052 resulted in limited effects on the innate immune response, rather than generalized immune suppression. In contrast, neutralization of TNF-α using a monoclonal antibody (TN3-19.12) resulted in immune suppression leading to loss of control of bacillary growth in the lungs of infected mice, with extensively increased pathology and greatly accelerated mortality. Anti-TNF-α monoclonal antibodies would not be expected to penetrate the plasma membrane, but rather act extracellular to bind and inactivate any available TNF-α. Since TNF-α is essential for orchestrating both the innate and acquired immune response, the complete neutralization of TNF-α would be expected to lead to a generalized immune suppression in the animal as seen here. Previous studies have shown that depletion of TNF-α by treatment of mice with anti-TNF-α antibodies leads to reactivation of latent *Mtb* infection [46], [47]. Similarly, treatment of patients with inflammatory disorders with the anti-TNF-α monoclonal antibody Infliximab has led to increased incidence of reactivation TB [48].

We also found that the combined effects of CC-3052 and INH resulted in a reduction in lung inflammation and accelerated resolution of lung pathology, as compared with mice treated with INH alone. TB is a striking example of TNF-α as a “double-edged sword”. TNF-α plays an essential role in controlling *Mtb* infection and disease severity, but this cytokine can also cause chronic inflammation leading to tissue damage [49], [50]. TB patients initiating antibiotic chemotherapy can experience a transiently increased inflammation and worsening of disease in association with an early burst in circulating levels of TNF-α [51]–[53]. From a clinical perspective, the limited impact of CC-3052 on TNF-α production by monocyte/macrophages is an advantage. The normal activation of T cells, which requires some TNF-α, ensured the control of bacillary growth via the contribution of the acquired immune response to protective immunity in CC-3052-treated animals. Thus, use of a PDE4i has enabled us to achieve this critical balance between limiting the negative effects of TNF-α, while retaining sufficient immunity to control infection. Recently, the multiple functions of TNF-α in granuloma formation and control of infection and disease pathogenesis were addressed using a computer-generated model, which predicted that a critical balance of TNF-α bioavailability must be maintained in order to achieve reduction of inflammation while retaining resistance to infection and microbial disease [54]. We propose that CC-3052 is a molecule that modulates TNF-α production, without adversely disrupting this critical balance, and can improve antibiotic-mediated bacillary clearance. Further studies are needed to evaluate the impact of PDE4 inhibition in a more clinically relevant TB regimen of multidrug therapy.

## Materials and Methods

### Ethics Statement

All animal experimentation was conducted following the Animal Welfare Act guidelines for housing and care of laboratory animals and performed in accordance with Public Health Service Policy Institutional regulations. Animal ethics approval for mouse infection with *M. tuberculosis* was obtained from the Institutional Animal Care and Use Committee at the Public Health Research Institute Center of University of Medicine & Dentistry, New Jersey (UMDNJ). PHRI Center ICPH IACUC approved our study protocol (Approval ID; 071D0810, Role of Immune Pressure in effectiveness of Tuberculosis Chemotherapy in the mouse model of TB).

### Chemicals and reagents

All chemicals were purchased from Sigma-Aldrich (St. Louis, MO), unless otherwise stated. PDE4 inhibitor CC-3052 was supplied by Celgene Corporation (Summit, NJ) and prepared as a suspension in sterile 0.2% carboxylmethylcellulose solution (CMC, Sigma). The purified monoclonal anti-TNF-α antibody (TN3-19.12) and isotype control hamster IgG1 antibody were provided by Dr. Robert D. Schreiber (Washington University in St. Louis, MO) [42].

### Infection of mice and drug treatment

Clinical *Mtb* strains CDC1551 and HN878 were grown to mid-log phase in Middlebrook 7H9 broth (Difco, Detroit, MI) containing 10% OADC enrichment (Beckton Dickinson, Franklin Lakes, NJ), 0.2% glycerol, and 0.05% Tween 80. Animals were housed in a Biosafety Level 3 pathogen-free facility. In brief, 8 to 10 week–old female B6D2F1 mice (Jackson Laboratory, Bar Harbor, ME) were infected with low dose (approximately 2 log_10_ cfu) of *Mtb* using an aerosol exposure system (CH Technology, Inc., Westwood, NJ) as described previously [55]. CC-3052 (25 mg/kg) was administered 5 days per week by gavage, and INH (50 mg/kg) was delivered ad libitum in drinking water. Neutralizing anti-TNF-α antibody TN3-19.12 (500 µg/mouse) and IgG1 isotype control (500 µg/mouse) were suspended in 300 µl of phosphate-buffered saline and administered by intraperitoneal injection once a week.

### CFU and body weight

The numbers of viable mycobacteria were evaluated using colony forming units (CFU) assay. For quantitation of initial inoculum, lungs were isolated from mice 3 hrs post-infection; lung homogenates were plated on 7H11 Middlebrook agar plates, incubated at 37°C for 21 days, and colonies counted. Bacterial loads are presented as the mean value in log_10_ ± standard deviation (SD). Body weight was measured every 3 days and expressed in percent (%) change to body weight at baseline (day 0). The Student's *t*-test was used to determine statistical significance between groups of data.

### Histopathology, immunohistology and morphometric analysis

Segments of lung tissue were fixed in 10% buffered formalin (Sigma-Aldrich) and paraffin-embedded. Sections were stained with Hematoxylin-Eosin (H&E) or Ziehl-Neelsen (ZN) acid-fast stain for evaluation of pathology and mycobacterial load, respectively. All photographs of the H&E and ZN stained sections were taken and analyzed using Nikon FX-35DX and microphot-FX. For morphometric analysis of granulomas in mouse lungs, H&E stained lung sections (n = 3–6 per group) were scanned with a PathScan Enabler IV scanner (Meyer Instruments, Houston, TX). SigmaScan Pro 5 software was used to count the number and size of lesions. The extent of lung involvement was calculated using three parameters: the average size of granulomas, the number of lesions per cm^2^ tissue, and the percent of the lung sections occupied by granulomas. Morphometric analysis was carried out by an independent single investigator blinded to the source of tissues. Immunohistochemistry staining was done with polyclonal anti-mouse CD3 antibody (Biocare Medical, Concord, CA). The number of CD3^+^ T cells was counted in 10×40 fields by 2 independent investigators blinded to tissue sources.

### DNA array and quantitative real time PCR

Global expression analysis of four independent arrays per group was performed using Affymetrix mouse genome ST 1.0 genes chips. For analysis, uninfected mice (baseline), untreated mice, CC-3052-treated mice at 14, 21, 28 and 42 days after infection (n = 4 per group) were euthanized. RNA from mouse lungs was isolated with Trizol (Invitrogen), DNase I treated, and further purified using RNeasy miniprep kit (Qiagen). Microarray experiments were performed once or twice and four independent arrays per group were analyzed. Data reduction and analysis of comparison of treatment groups was normalized, and filtered expression files were analyzed using Partek Genomic Suite. SAM (significance analysis of microarray) tool was used for adjusting the *P* values from a comparison test based on the number of tests performed. Differentially expressed genes were selected with a cutoff value of ±1.5 fold change (P<0.05). Gene ontology analysis was performed using Pathway Express (Wayne State University, Detroit, MI). Reverse transcription of total lung RNA for the qPCR was completed with a first-strand cDNA synthesis kit by oligo (dT) priming (Invitrogen), according to the manufacturer's protocol. Real time PCR was performed using the Invitrogen SYBR GreenER qPCR universal module, according to the manufacturer. Primers specific to genes of interest were designed as recommended by PrimerBank (www.primerbank.org). Quantified, purified and diluted PCR product was used to generate external standard curves for each primer pair. Cycle number values were converted to copy number using these curves post-amplification. qPCR of glyceraldehyde-3-phosphate dehydrogenase (*gapdh*) was always run in parallel with that of the other genes of interest and levels of gene expression measured were normalized to the housekeeping gene *gapdh*. The complete gene lists are accessible through GEO database, NCBI accession number GSE25313.

### Single tissue cell preparation

Single cell suspensions from lung tissue were prepared by incubating minced tissue with collagenase A (1 mg/ml; Boehringer-Mannheim) and DNase I (25–50 U/ml; Sigma-Aldrich) for 1 hour at 37°C. The digested lung was further disrupted by gentle teasing through a cell strainer (BD Bioscience). Spleens were disrupted by teasing the tissue directly through a cell strainer without digestion. Red blood cells were lysed with ACK buffer (GIBCO), washed and resuspended in RPMI 1640 medium supplemented with 10% fetal calf serum (FCS), penicillin/streptomycin (50 U/50 µg/ml; R10). Total number of cells was determined by microscopic counting using a hemocytometer. Spleen cells were cultured at 37°C at a density of 2.5×10^5^/100 µl in 96-well U-bottom plates. The cells were stimulated in R10 with Purified Protein Derivative (PPD, 10 µg/ml final; Staten Serum Institute) or lipopolysaccharide (LPS, 1 µg/ml final; Sigma) and incubated at 37°C for 6 hrs, 24 hrs or 6 days. At each time point, culture supernatant was removed, filtered using 0.22 µm low-binding microfuge filter tubes (Millipore, Billerica, MA) and stored at −80°C for cytokine assays. Isolated cells were frozen at −80°C in R10 with 10% DMSO for flow cytometric analysis.

### Flow cytometric analysis of cell surface markers and intracellular cytokine assay

Spleen or lungs were removed from CC-3052 treated or untreated infected mice at the indicated time points and processed as described [56]. Briefly, cells were stained with FITC-anti-CD14, PE-anti-CD4, PercP-anti-CD3, and APC-anti-CD8 monoclonal antibodies (BD Bioscience). For detection of intracellular TNF-α, spleen cells, cultured in the presence of PPD (10 µg/ml final) for 6 hrs, were stained with PE-anti-IFN-γ, ACP-anti-TNF-α, FITC-anti-CD4, PercP-anti-CD3, APC-anti-CD8 and FITC-anti-CD14 (BD Biosciences, San Jose, CA). Intracellular IFN-γ in lung cells was measure without prior in vitro stimulation. Data acquisition and analysis were performed using FACSCalibur (BD Bioscience) and FlowJo software (Tree Star, Ashland, OR) respectively.

### Proliferation assay

Spleen cells isolated as described above were stained with carboxyfluorescein succinimidyl ester dye (CFSE; Invitrogen, Carlsbad, CA), according to manufacturer's instructions. 2.5×10^5^ CFSE-labeled cells/100 µl were plated in 96-well U-bottom plates, and stimulated for 6 days with ConA (5 µg/ml final concentration; Sigma) or heat-killed, sonicated *Mtb* CDC1551 at a multiplicity of infection of 10 to 1 (MOI 10∶1), or left unstimulated. Cells were fixed and stored at −80°C. Cells were stained for surface markers with the following mAbs: PE-anti-CD4, APC-anti-CD8 and PerCP-anti-CD3 (BD Bioscience). Data acquisition and analysis were performed using FACSCalibur (BD Bioscience) and FlowJo software (Tree Star, Ashland, OR). Proliferation was measured as a reduction in CFSE fluorescence intensity.

### Statistical analysis

All data are presented as the mean ± standard deviation (SD). Statistical significance was determined using SAM (significance analysis microarrays) and Student's *t-*test. A value of *P*<0.05 was considered significant.

### Accession numbers

Detailed information for the genes/proteins from this study can be found at NCBI GenBank database www.ncbi.nih.gov/genbank. The gene name and ID numbers in this publication are listed in Table S1. The ID numbers of TNF-α and IFN-γ shown in this publication are NP_038721 and AAI19061, respectively.

## Supporting Information

Figure S1 **Morphometric analysis of the extent of the granulomatous infiltration in the lungs.** H&E stained lung sections from Mtb-infected mice with or without CC-3052 and/or INH treatment at 35 or 63 days post-infection. The extent of the lung tissue involved in granuloma formation was represented as (A) the number of granulomas per cm^2^ and (B) the percentage of lung involved. Results shown are mean ± SD from 3–4 mice per group per time point.
(TIF)

Figure S2 **Phenotype of lung cells isolated from infected mice.** Lung cells from mice infected for 28 days, with or without CC-3052 treatment, were stained for T cell and macrophage markers and analyzed by flow cytometry. (A) Representative dot and contour plots. (B) Percentage of viable CD4^+^, CD8^+^ and CD14^+^ cells in the lungs. Data are expressed as the mean ± SD of 8 replicates per untreated mice and 8 replicates per CC-3052 treated mice. (C) IFN-γ producing CD4^+^ and CD8^+^ cells (right panel). Data are expressed as the mean ± SD of 2 mice per group.
(TIF)

Table S1 Differentially expressed mouse genes by CC-3052 treatment. Gene expression was analyzed from total RNA of uninfected and *Mtb*-infected mouse lungs, treated or not treated with CC-3052, at 28 days post-infection. Results are representative of 4 independent arrays per group and presented as relative gene expression. Fold change (≥1.5 fold) of differentially expressed genes from arrays was statistically significant based on SAM analysis (*P<*0.05). The gene name and ID numbers are available at www.ncbi.nih.gov/genbank.
(TIF)
