# Supplementary figures and images for: Phosphodiesterase 4 Inhibition Reduces Innate Immunity and Improves Isoniazid Clearance of Mycobacterium tuberculosis in the Lungs of Infected Mice
Source: PLoS One. 2011 Feb 25;6(2):e17091. doi: 10.1371/journal.pone.0017091 (PMC3045423; doi:10.1371/journal.pone.0017091)

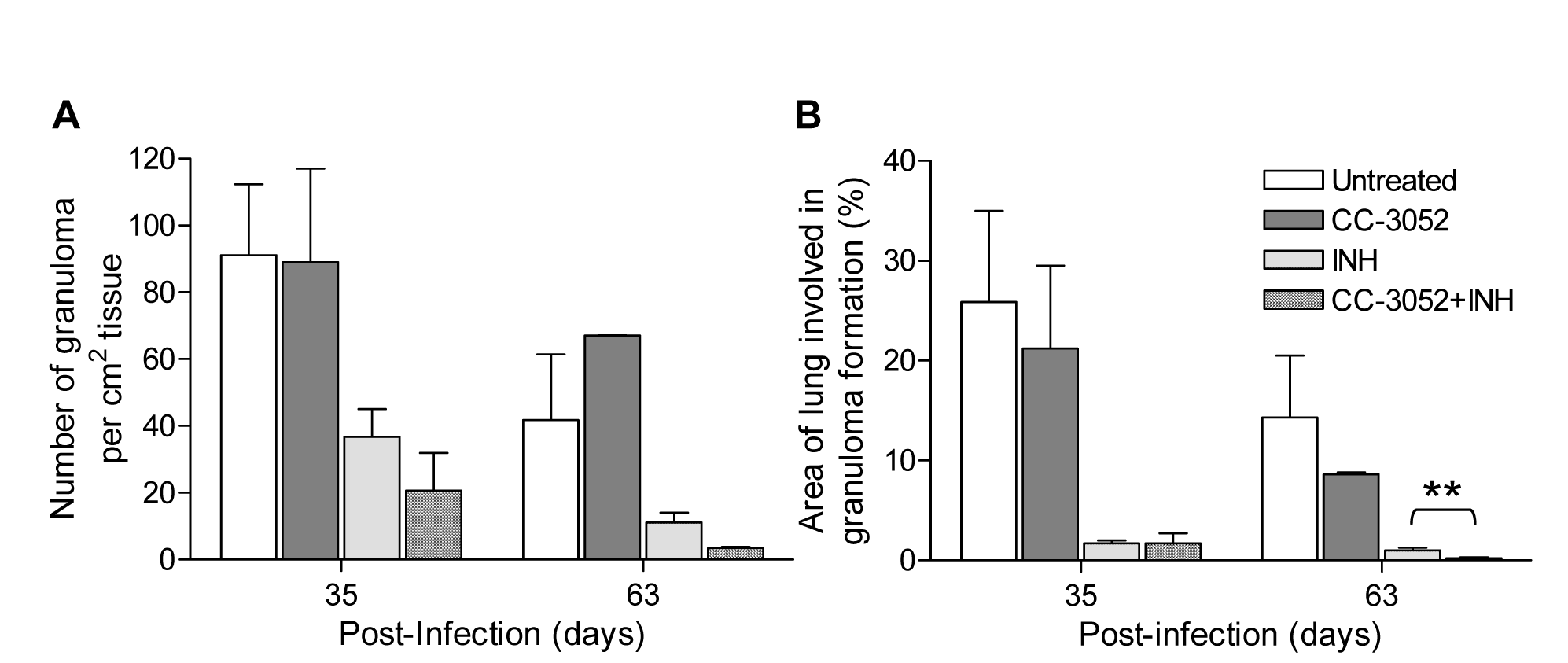

Supplement: Figure S1 — Morphometric analysis of the extent of the granulomatous infiltration in the lungs. H&E stained lung sections from Mtb-infected mice with or without CC-3052 and/or INH treatment at 35 or 63 days post-infection. The extent of the lung tissue involved in granuloma formation was represented as (A) the number of granulomas per cm2 and (B) the percentage of lung involved. Results shown are mean ± SD from 3–4 mice per group per time point. (TIF) [file pone.0017091.s001.tif]

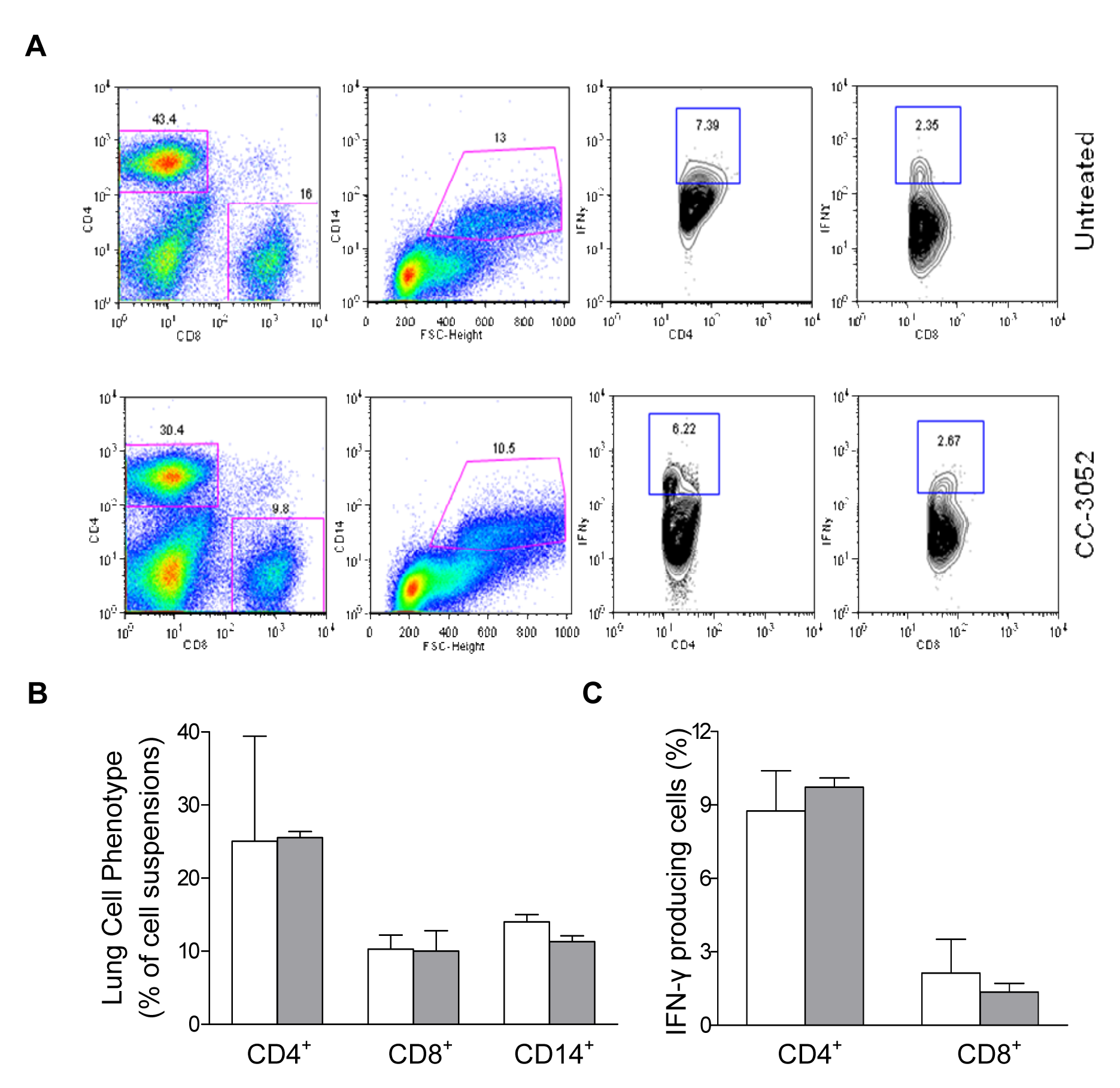

Supplement: Figure S2 — Phenotype of lung cells isolated from infected mice. Lung cells from mice infected for 28 days, with or without CC-3052 treatment, were stained for T cell and macrophage markers and analyzed by flow cytometry. (A) Representative dot and contour plots. (B) Percentage of viable CD4+, CD8+ and CD14+ cells in the lungs. Data are expressed as the mean ± SD of 8 replicates per untreated mice and 8 replicates per CC-3052 treated mice. (C) IFN-γ producing CD4+ and CD8+ cells (right panel). Data are expressed as the mean ± SD of 2 mice per group. (TIF) [file pone.0017091.s002.tif]

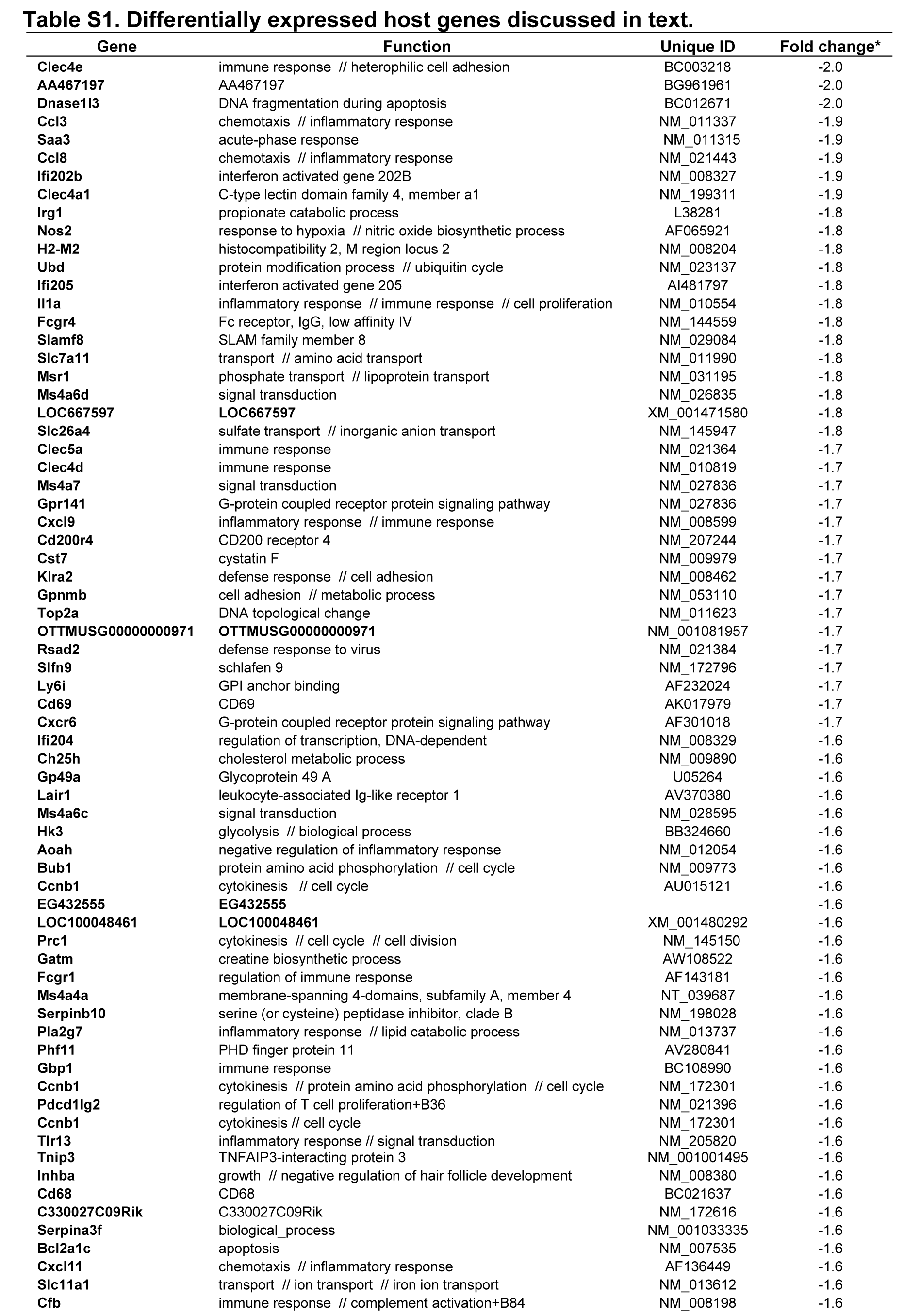

Supplement: Table S1 — Differentially expressed mouse genes by CC-3052 treatment. Gene expression was analyzed from total RNA of uninfected and Mtb-infected mouse lungs, treated or not treated with CC-3052, at 28 days post-infection. Results are representative of 4 independent arrays per group and presented as relative gene expression. Fold change (≥1.5 fold) of differentially expressed genes from arrays was statistically significant based on SAM analysis (P<0.05). The gene name and ID numbers are available at www.ncbi.nih.gov/genbank. (TIF) [file pone.0017091.s003.tif]
